# Toward the Impact of Job Satisfaction and Collective Efficacy on EFL Teachers’ Professional Commitment

**DOI:** 10.3389/fpsyg.2022.938125

**Published:** 2022-07-07

**Authors:** Fan Zhang

**Affiliations:** College of Foreign Languages, Xi’an University of Finance and Economics, Xi’an, China

**Keywords:** professional commitment, job satisfaction, collective efficacy, EFL teachers, educational system

## Abstract

Since the success of any educational system is tied to the teachers’ professional commitment, discovering the determinants of this construct seems vital. In line with this, a huge number of inquiries have evaluated the effects of personal, contextual, and professional variables on teachers’ professional commitment. However, the impacts of job satisfaction and collective efficacy have remained unclear. Against this backdrop, the current review article seeks to theoretically explain the impacts of these constructs on EFL teachers’ professional commitment using the available documents. The review findings illuminated that EFL teachers’ professional commitment heavily relies on their job satisfaction and collective efficacy beliefs. The implications for educational principals and teacher educators are finally discussed.

## Introduction

Teachers in any educational system are perceived as the main pillars of education in that without their presence and continual efforts, pupils will find nothing but failure. Given the prominent role of teachers in educational settings, how deeply they are committed to their profession seems critical (Yu et al., 2021). Individual teachers’ commitment to their profession is called “professional commitment,” referring to their psychological and emotional attachment to the teaching vocation, pupils, and colleagues (Lee et al., 2011). Reyes (1990) conceptualized teacher professional commitment as “the relative strength of an individual teacher’s identification with and involvement in a particular educational context” (cited in Chan et al., 2008, p. 598). To underline the significance of teacher professional commitment, Moses et al. (2017) articulated that the degree to which teachers devote themselves to their careers can enormously influence their effectiveness. Wang et al. (2021) took one step further by declaring that teachers’ professional success is subjective to their professional commitment. To them, committed teachers who are psychologically attached to teaching are more likely to succeed in their profession. In a similar vein, Altun (2017) noted that commitment to the teaching profession leads teachers to devote themselves to fulfilling their job-related responsibilities, which may result in increased learning outcomes. Taken together, teacher professional commitment is tied to teacher success (Wang et al., 2021), increased teaching effectiveness (Moses et al., 2017), and improved learning outcomes (Altun, 2017). Because of this, exploring the antecedents and predictors of teacher professional commitment appears to be necessary. In line with this necessity, remarkable academic endeavors have been directed toward discovering the determinants of this construct (e.g., Aliakbari and Amoli, 2016; Han et al., 2016; Lambersky, 2016; Hallinger et al., 2018; Liu, 2019; Demir, 2020; Zheng et al., 2020, among others). Nonetheless, relatively limited attention has been given to identifying the role of job satisfaction and collective efficacy in teachers’ professional commitment.

The notion of job satisfaction has been broadly defined by Spector (1997) as a favorable emotional state caused by one’s evaluation of his or her profession and professional experiences. Likewise, teacher job satisfaction pertains to individual teachers’ contentment with the teaching profession and instructional experiences (Soodmand Afshar and Doosti, 2016). Teachers’ job satisfaction is believed to be negatively linked with their intention to quit, emotional exhaustion, and burnout (Skaalvik and Skaalvik, 2011; Larkin, 2015; Esfandiari and Kamali, 2016; Wang and Guan, 2020). Skaalvik and Skaalvik (2011), for instance, maintained that teachers who are happy with their profession, workplace, and working conditions are less likely to experience emotional exhaustion. Besides, this notion has also been confirmed by Wang and Guan (2020) that teachers’ demotivation or disengagement in their job will be contagious to their students. However, those teachers who have a strong sense of belonging to teaching are more prone to pursuing their vocation. Esfandiari and Kamali (2016) also declared that teachers with high levels of job satisfaction are less prone to burnout. To them, being satisfied with the teaching profession and its conditions not only prevents burnout but also drives teachers to constantly participate in their workplaces.

The construct of collective efficacy has been generally conceptualized by Bandura (1997) as “a group’s shared belief in its conjoint capabilities to organize and execute the courses of action required to produce given levels of attainments” (cited in Goddard and Salloum, 2011, p. 644). Within an educational environment, collective efficacy refers to teachers’ strong belief in faculty members’ capability to promote students’ learning outcomes (Klassen et al., 2010). As Goddard et al. (2015) pointed out, teachers’ collective efficacy represents their viewpoints of “group-level attributes”; that is, assessments of the capacities of the group or educational institution to which they belong. Teachers’ collective efficacy beliefs are perceived to be remarkably affected by “past success,” “observation of other groups’ successes,” and “encouragement from influential others” (Klassen et al., 2014; Han and Wang, 2021). According to Khong et al. (2017), teachers with strong collective efficacy beliefs can make a substantial difference in students’ academic performance. In a similar vein, Lu and Mustafa (2021) articulated those teachers who possess a higher degree of collective efficacy can effectively engage students in the learning process. It is largely because teachers who have faith in faculty members’ teaching abilities are more inclined to participate in their workplaces. Teachers’ active participation, according to Zhang and Yang (2021), encourages pupils to take an active role in instructional-learning environments.

Because of the significance of teachers’ job satisfaction and collective efficacy, numerous scholars have taken their educational consequences into consideration. A group of researchers has explored the positive effects these constructs may have on students’ academic behaviors (Wang et al., 2021; Gao et al., 2022). The rest have investigated their impact on teachers’ personal and professional behaviors. Nevertheless, the influences of these variables on teachers’ professional commitment have rarely been studied (Akpan, 2013; Bashir, 2017; Bashir and Gani, 2020; Cansoy et al., 2020) in ESL/EFL educational context. Additionally, the role of job satisfaction and collective efficacy in teachers’ commitment has not been addressed in any review article. To address this lacuna, the present review study aims to describe the impacts of EFL teachers’ job satisfaction and collective efficacy on their professional commitment.

### Job Satisfaction

Job satisfaction generally refers to the state of being satisfied with a particular occupation, its environment, and its conditions (Zhu, 2013). Teacher job satisfaction in this respect pertains to “teachers’ affective reactions to their work or to their teaching role” (Skaalvik and Skaalvik, 2015, p. 183). As put forward by Zeinabadi (2010), teacher job satisfaction has to do with how positively an individual teacher assesses his or her vocation and vocational condition. In line with this, Bogler and Nir (2012) articulated that job satisfaction involves two major dimensions, including “*intrinsic job satisfaction*” and “*extrinsic job satisfaction*.” Intrinsic job satisfaction refers to the degree of satisfaction an individual teacher receives from the nature of the profession (Lopes and Oliveira, 2020). Extrinsic job satisfaction, on the other hand, pertains to the degree of satisfaction an individual teacher receives from the working conditions (Rezai et al., 2021).

### Collective Efficacy

The term “collective efficacy” generally refers to “the perceived performance capability of a social system as a whole” (Dimopoulou, 2014, p. 1471). More specifically, teacher collective efficacy involves teachers’ perceptions of their own and their colleagues’ potency to significantly improve learners’ academic outcomes (Guidetti et al., 2018; Han and Wang, 2021). As noted by Fathi and Savadi Rostami (2018), teachers’ collective efficacy deals with their viewpoint, appraisal, endeavor, perseverance, and inclination to stay together. Teachers’ collective efficacy beliefs are believed to be directly affected by the transformational (Ninkovic and Knezevic, 2018) and instructional leadership (Cansoy and Parlar, 2018) of educational principals.

### Professional Commitment

The concept of professional commitment, also called occupational commitment, pertains to “the depth and strength of the attachment between an employee and his/her occupation” (Ibrahim and Iqbal, 2015, p. 36). In this sense, teacher professional commitment refers to how strongly teachers are attached to teaching profession (Qin, 2021). As a multidimensional variable, teacher professional commitment comprises three main facets, namely *“affective commitment,” “normative commitment,”* and *“continuance commitment”* (Wang et al., 2021). As the first facet, affective commitment refers to the emotional bond that exists between an individual teacher and the teaching vocation. The second facet, normative commitment, relates to an individual teacher’s inclination to pursue the teaching profession due to moral considerations. The last facet, continuance commitment, pertains to teachers’ proclivity to stay in the teaching profession due to the professional relationships they have with their colleagues, pupils, and educational principals (Ganjali et al., 2020).

### The Impact of Job Satisfaction and Collective Efficacy in EFL Teachers’ Professional Commitment

The favorable effect of job satisfaction on EFL teachers’ professional commitment can be illustrated by what Gilbert et al. (2014) declared in this respect. They maintained that those who are happy and content with the teaching profession and its working conditions are emotionally and psychologically attached to their vocation. To them, job satisfaction as a driving force prompts teachers to become committed to their profession. Similarly, regarding positive psychology assumptions, Buettner et al. (2016) stated that positive emotional states such as job satisfaction inspire teachers to devote themselves to the teaching profession. Besides, the positive impact of collective efficacy on EFL teachers’ commitment can be readily justified through what Skaalvik and Skaalvik (2019) articulated regarding the value of teachers’ individual and collective efficacy beliefs. They noted that teachers who have faith in their own and their coworkers’ abilities are commonly more committed to their profession. It is because individual and collective efficacy beliefs empower them to confidently participate in their workplaces (Minghui et al., 2018).

## Empirical Evidence

As previously mentioned, the impacts of job satisfaction and collective efficacy on teachers’ professional commitment have seldom been examined (Akpan, 2013; Bashir, 2017; Bashir and Gani, 2020; Cansoy et al., 2020). Akpan (2013), for example, examined the extent to which Nigerian EFL teachers’ professional commitment may be affected by their job satisfaction. To do so, two pre-designed questionnaires were given to 290 EFL teachers. Using regression analysis, the researcher found that teachers’ job satisfaction can greatly affect their professional commitment. In another study, Bashir and Gani (2020) also evaluated the effects of teachers’ job satisfaction on their commitment. To accomplish this, 396 instructors were invited to fill out two close-ended questionnaires. Performing structural equation modeling, the scholars found that teachers’ commitment is subjective to their job satisfaction. They reported that the higher the job satisfaction, the stronger the teachers’ professional commitment. In their study, Cansoy et al. (2020) studied collective teacher efficacy in association with teacher commitment. To do this, the reliable scales of the variables were administered to 247 teachers. The results of correlational tests demonstrated a positive correlation between teachers’ collective efficacy and their commitment.

## Conclusion and Pedagogical Implications

The definitions, facets, and core components of job satisfaction, collective efficacy, and professional commitment were thoroughly addressed in this review. Further, using available documents, the impacts of EFL teachers’ job satisfaction and collective efficacy on their professional commitment were discussed. Taking the existing documents into account, it seems plausible to conclude that EFL teachers’ professional commitment highly depends on their job satisfaction and collective efficacy beliefs. This appears to be enlightening and instructive for educational principals and teacher educators. It is deemed useful for educational principals in that they can considerably promote teachers’ job satisfaction by providing them with appropriate working conditions. The finding is also perceived to be beneficial for teacher educators as they can improve their student teachers’ collective efficacy beliefs. To do so, they need to equip their student teachers with sufficient instructional knowledge.

## Author Contributions

The author confirms being the sole contributor of this work and has approved it for publication.

## Conflict of Interest

The author declares that the research was conducted in the absence of any commercial or financial relationships that could be construed as a potential conflict of interest.

## Publisher’s Note

All claims expressed in this article are solely those of the authors and do not necessarily represent those of their affiliated organizations, or those of the publisher, the editors and the reviewers. Any product that may be evaluated in this article, or claim that may be made by its manufacturer, is not guaranteed or endorsed by the publisher.
